# No More Venous Ulcers—What More Can We Do?

**DOI:** 10.3390/jcm12196153

**Published:** 2023-09-23

**Authors:** Agata Stanek, Giovanni Mosti, Temirov Surat Nematillaevich, Eva Maria Valesky, Tanja Planinšek Ručigaj, Malika Boucelma, George Marakomichelakis, Aaron Liew, Bahar Fazeli, Mariella Catalano, Malay Patel

**Affiliations:** 1 Department of Internal Medicine, Angiology and Physical Medicine, Faculty of Medical Sciences in Zabrze, Medical University of Silesia, Batorego 15 Street, 41-902 Bytom, Poland; 2 Vascular Independent Research and Education, European Foundation, 20157 Milan, Italy; gmarakom@gmail.com (G.M.); aaron.liew@nuigalway.ie (A.L.); bahar.fazeli@gmail.com (B.F.); mariella.catalano@unimi.it (M.C.); drmalaypatel@gmail.com (M.P.); 3 VAS-International Consortium—International No More Venous Ulcers Strategic Network, 20157 Milan, Italy; giovanni.mosti10@gmail.com (G.M.); temirovs@gmail.com (T.S.N.); eva.valesky@kgu.de (E.M.V.); t.rucigaj@gmail.com (T.P.R.); mboucelma@yahoo.fr (M.B.); 4 Angiology Department, MD Barbantini Clinic, Via del Calcio 2, 55100 Lucca, Italy; 5 Department of Specialized Surgery, Central Hospital of Ministry of Internal Affairs, Chimboy St. 2 A, Almazar District, Tashkent 100095, Uzbekistan; 6 Department of Dermatology, Venereology and Allergology, University Hospital, Goethe University Frankfurt, Theodor-Stern-Kai 7, 60596 Frankfurt am Main, Germany; 7 Dermatovenereological Clinic, University Medical Centre Ljubljana, Gradiskova 10 Street, 1000 Ljubljana, Slovenia; 8 Department of Internal Medicine, University of Algiers, Bachir Mentouri Hospital, Algiers 16208, Algeria; 9 4th Department of Internal Medicine, General Hospital of Evangelismos, 16676 Athens, Greece; 10 Department of Medicine, Portiuncula University Hospital, University of Galway, H91 TK33 Galway, Ireland; 11 Support Association of Patients of Buerger’s Disease, Buerger’s Disease NGO, Mashhad 9183785195, Iran; 12 Department of Biomedical, Clinical Sciences L Sacco Hospital, Inter-University Research Center on Vascular Disease, University of Milan, 20157 Milan, Italy; 13 Department of Vascular Surgery, Apollo CVHF, Heart Institute, Ahmedabad 380059, India

**Keywords:** venous ulcer, recurrent venous ulcer, compression therapy, conservative treatment, invasive treatment, costs, prevention, burden of illness

## Abstract

Venous leg ulcers (VLUs) are the most severe complication caused by the progression of chronic venous insufficiency. They account for approximately 70–90% of all chronic leg ulcers (CLUs). A total of 1% of the Western population will suffer at some time in their lives from a VLU. Furthermore, most CLUs are VLUs, defined as chronic leg wounds that show no tendency to heal after three months of appropriate treatment or are still not fully healed at 12 months. The essential feature of VLUs is their recurrence. VLUs also significantly impact quality of life and could cause social isolation and depression. They also have a significant avoidable economic burden. It is estimated that the treatment of venous ulceration accounts for around 3% of the total expenditure on healthcare. A VLU-free world is a highly desirable aim but could be challenging to achieve with the current knowledge of the pathophysiology and diagnostic and therapeutical protocols. To decrease the incidence of VLUs, the long-term goal must be to identify high-risk patients at an early stage of chronic venous disease and initiate appropriate preventive measures. This review discusses the epidemiology, socioeconomic burden, pathophysiology, diagnosis, modes of conservative and invasive treatment, and prevention of VLUs.

## 1. Introduction

### 1.1. Definition of Venous Leg Ulcer

According to the CEAP classification, revised in 2004, a venous leg ulcer (VLU) is defined as a full-thickness skin defect, most frequently in the lower leg and ankle region, that fails to heal spontaneously and is sustained by venous hypertension due to chronic venous disease [1]. VLUs usually occur at the malleolar part on the medial and lateral sides of the ankle. However, they also may appear on the supra-malleolar and infra-malleolar areas of the leg and foot [2]. Furthermore, most VLUs are chronic leg ulcers (CLUs), defined as chronic leg wounds that show no tendency to heal after three months of appropriate treatment or are still not fully healed at 12 months [3]. Moreover, it is estimated that between 40% and 50% remain active between 6 and 12 months, and that 10% remain active up to 5 years [4]. It also has been shown that a VLU that fails to decrease in size by 30% (percentage area reduction) of its initial size over the first four weeks of treatment has a 68% probability of failing to heal within 24 weeks [5].

### 1.2. Epidemiology and Socioeconomic Burden

VLUs are the most severe complication caused by the progression of chronic venous insufficiency (CVI) [1,6,7]. They account for approximately 70%–90% of all chronic leg ulcers [8]. Women suffer three times more than men, and the incidence increases with age [9], and 40% of patients with VLUs present with underlying deep venous disease (DVD) [10].

O’Meara et al. say that 1% of the Western population sometimes suffers from a VLU [11]. Other population-based studies have reported similar prevalence rates, ranging from 0.7% to 2.7% [12]. VLUs across the globe are expected to rise due to an aging and increasingly overweight population.

The essential feature of VLUs is their recurrence. In the study conducted by Finlayson et al., the average relapse time was 42 weeks, with incidences of 22% at three months, 39% at six months, 57% at 12 months, 73% at two years, and 78% within a 3-year follow-up [6]. Moreover, it has been shown that deep venous disease-related VLUs (DRVs) are thought to have higher recurrence rates and an even more significant economic burden. In the study conducted by Kollour et al., the total annual incidences of new or recurrent DRVs in Australia, France, Germany, Italy, Spain, the U.K., and the U.S. were estimated at 122,000, 263,000, 345,000, 253,000, 85,000, 230,000, and 643,000 events, respectively, in 2019 [10].

Additionally, VLUs are related to a significant cause of morbidity. They also have an enormous impact on the social burden. It has been reported that 4.6 million workdays per year are lost secondary to CVI, in which VLUs are the most severe complication [12]. VLUs also significantly decrease quality of life and are painful [13]. Moreover, they could even cause social isolation and depression [14].

Apart from the substantial social burden, VLUs also significantly impact the economic burden. It has been estimated that treating venous ulceration accounts for around 3% of the total expenditure on healthcare [15]. An analysis of Secure Anonymized Information Linkage Databank data from 2007 to 2017, including approximately 2.5 million people over 11 years, showed that the direct cost of managing patients with VLUs was GBP 7706 per patient per annum, which translated to an annual cost of over GBP 2 billion when extrapolated to the UK population [16]. In the U.S., in 2019, the annual direct medical costs for patients with DRVs managed conservatively were approximately USD 10.73 billion (USD) or USD 5527 per person per year [10]. In Singapore (2017), the estimated gross healthcare cost per patient for inpatient, specialist outpatient, and primary care for VLUs was USD 16,761 for one year [17]. In Australia (2014), the total direct costs of treating VLUs (in public and private hospitals and residential care settings) were estimated to be USD 802.55 million [18]. In Germany (2007), the mean total cost of the ulcer per year and patient was EUR 9569 [19]. Guest et al., in their study, noticed that the patient care cost of an unhealed wound was 135% more than that of a healed wound. The authors concluded that the clinical and economic benefits to patients and the NHS could accrue from three strategies focusing on wound prevention, accurate diagnosis, and improving wound-healing rates [20].

A large International Collaborative Network of Scientific Societies (International No More Venous Ulcers Strategic Network) identified common education and awareness strategies to help prevention, intending to join expertise and energies worldwide in concrete plans. Coordination between the existing action plans by different organizations would be an effective way to follow in the future.

This review aims to present the current knowledge on the pathophysiology and treatment of VLUs and, furthermore, to consider what more can be done to decrease their number. 

## 2. Pathophysiology of Venous Ulcers

Patients with venous leg ulcers have venous hypertension and abnormally sustained venous pressure elevation upon ambulation (normal venous pressure decreases with walking), which results from vein reflux or outflow obstruction.

Venous outflow may also be impaired due to poor calf muscle pump function, which damages the venous system’s ability to overcome the venous blood return to the heart. The limitation of ankle movement seems to be an essential contributor to calf muscle pump failure and a risk factor for ulceration [21]. Several risk factors of VLU development have been identified, including, among others, an age older than 55 years, an increased body mass index (BMI), a family history of CVI, a history of venous thromboembolism disease and superficial thrombophlebitis, a sedentary lifestyle, skeletal or joint disease of the legs, multiparous women, and severe stages of chronic venous diseases (lipodermatosclerosis, active or healed venous ulcers in history) [22]. 

Although venous hypertension results in ulceration, the exact mechanism remains unclear. Several hypotheses have been proposed, such as abnormalities of the fibrinolytic system, peripapillary fibrin deposition causing decreased oxygen diffusion to tissues, the trapping of growth factors by extravasated macromolecules around the vessels and in the dermis, limiting their function, and leucocyte margination and activation with the subsequent local release of inflammatory mediators [23]. Consequent microcirculatory changes lead to venous hypertensive microangiopathy (enlarged permeable capillaries, abnormally increased skin flux, edema, altered microlymphatic circulation, decreased partial pressure of oxygen, and increased carbon dioxide) that results in ulceration [24].

The effect on the microcirculation begins with altered shear stress on the endothelial cells, causing them to release vasoactive agents and express selectins, inflammatory molecules, chemokines, and prothrombotic precursors [25,26]. Mechanical forces and low shear stress are sensed by the endothelial cells via intercellular adhesion molecules-1 (ICAM1, CD 54), vascular cell adhesion molecules1 (VCAM-1, CD-106), and endothelial leucocyte adhesion molecule1 (CD-62, E-selectin). CVD patients have increased expressions of ICAM-1 and VCAM-1. A key component of inflammation in VLUs is the increased expressions of matrix metalloproteinases and the production of cytokines (Transforming growth factor-β1, Tumor necrosis factor-α, Interleukin-1) [27]. 

Another area of research in the pathophysiology of LVUs is gap junctions. Gap junctions are proteins that play critical roles in the pathogenesis of chronic wounds, mainly involved in inflammation, edema, and fibrosis. Connexins (a component of gap junctions) are abnormally elevated in the wound margins of VLUs. Connexins seem to play an essential role in the inflammatory response and VLU healing [28].

**Figure 1:**
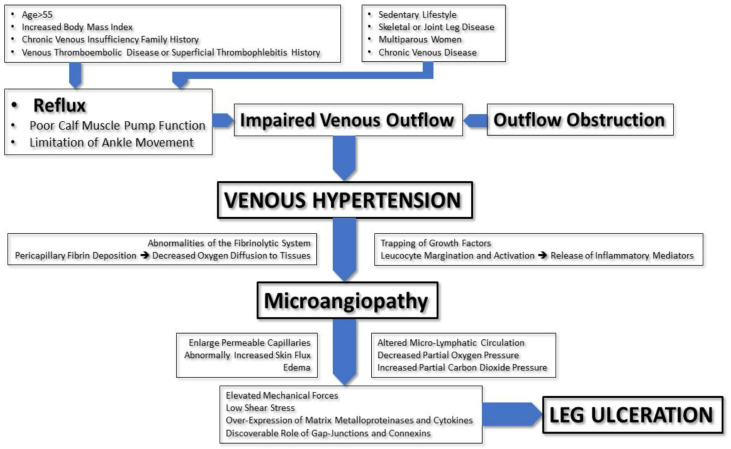
Pathophysiology of venous ulcers.

The pathophysiology of venous ulcers is shown in Figure 1. 

## 3. Diagnosis of Venous Ulcers

The diagnosis of leg ulcers is based on medical history, clinical assessments, functional/diagnostics testing, blood tests, biopsy, bacteriologic or mycologic swabs, histopathology, and direct immunofluorescence [29,30,31,32] (Table 1). The clinical diagnosis as the first step is available everywhere, allowing for the diagnosis of the majority of VLUs. In the next step, several complementary diagnostic procedures are used [27,29] (Table 2). 

Ulcers on the lower extremities occur from various etiologies; it is essential to know the possible differential diagnoses of the various wounds on the lower leg. The clinical findings may be similar, but the etiologies can differ [30,32] (Table 2). 

Regarding investigations, individual studies’ recommendations could be more robust [25,26] (Table 3).

VLUs are more common in patients with positive family histories of chronic venous insufficiency, in patients with higher body mass indexes, in patients with histories of pulmonary embolism or superficial-/deep-vein thrombosis, in diseases of the skeleton or joints of the lower extremities (due to poor mobility or immobility of the ankle), in women with multiple pregnancies or on hormone therapy, in those patients who have previously had ulcers, in patients who have lipodermatosclerosis and other signs of venous disease or venous insufficiency, and in patients who have typical evening swelling of the legs. Poor prognostic signs for healing are an ulcer duration longer than three months, an initial ulcer size of 10 cm or more at the start of treatment, and the presence of lower-extremity arterial disease. Patients with venous ulcers have ulcers that are usually shallow with well-defined edges and that are often located in the lower two-thirds of the lower leg. Signs of venous disease, such as varicose veins, edema, corona phlebectatica, atrophie blanche, and eczema due to stasis, may also be present. At the same time, patients should have palpable pedal pulses and a calculated brachial–ankle index above 0.85. On Duplex ultrasound, superficial and/or deep veins or perforating veins are insufficient. Severe complications include infection and malignant changes in the ulcer. All these symptoms and signs, as well as associated diseases and specifics in the anamnesis and clinical picture, as well as objections to investigations, help us diagnose venous ulcers and distinguish them from other possible causes of leg ulcers [33,34,35].

## 4. Current Treatment of Venous Ulcers

Achieving the goal of having no more VLUs would prevent their formation. Prevention by conservative methods would be primary or secondary. Unfortunately, we have very few negative data for primary prevention. Indeed, only one paper reported that medical compression stockings (MCSs) could not prevent ulcer formation in patients with previous deep-vein thrombosis [36]. However, more data confirm that MCSs can effectively prevent ulcer recurrence. It is also essential to have less VLUs, as ulcer recurrence is up to about 78% of patients with histories of previous VLUs [37] in the first few months after ulcer healing [38,39]. The compression pressure must be as high as possible, as it was shown that the higher the compression pressure, the lower the risk of VLU recurrence [40]. 

Nevertheless, patient compliance must be considered, as it appears to be even more critical than the MCS compression pressure. Patients wearing compression are more likely to avoid ulcer recurrence than patients not wearing compression independent of the MCS pressure [41]. Even if this chapter deals with conservative treatment, it is worthwhile highlighting that surgical ablation is more effective than compression alone in preventing ulcer recurrence [42].

While it is important to have fewer VLUs, trying to shorten the ulcer healing time is as meaningful too. Both surgical and conservative procedures are effective at shortening the healing time. It has been proven that invasive procedures are more effective than compression alone to shorten the VLU healing time [43]. 

**Figure 2:**
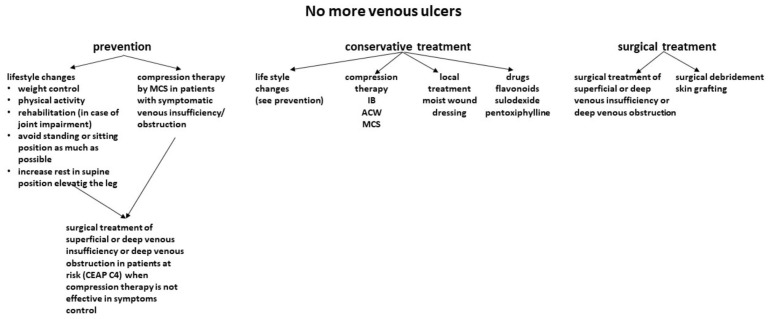
Different modes of prevention and treatment of venous leg ulcers (CEAP C4—skin damage due to chronic insufficiency; IB—inelastic bandage; ACW—adjustable compression wrap; MCS—medical compression stocking.

The different modes of the prevention and treatment of VLUs are shown in Figure 2.

### 4.1. Conservative Treatment

Conservative treatment of VLU patients mainly includes compression therapy, lifestyle changes, physical rehabilitation, local treatment, and pharmacologic treatment.

#### 4.1.1. Compression Therapy

VLUs are primarily due to a venous hemodynamic impairment characterized by ambulatory venous hypertension. Any VLU treatment aims to reduce ambulatory venous hypertension to favor VLU healing. Surgical or conservative procedures can achieve ambulatory venous hypertension reduction. Compression therapy is considered the first treatment option in the conservative management of VLUs. It is the only therapeutical procedure that achieved grade 1A in the most recent guidelines and consensus papers on VLU treatment [44,45,46,47]. Compression therapy can be exerted using inelastic materials (short-stretch or inextensible bandages, adjustable compression wraps (ACWs), and hybrid pumps) and elastic fabrics (stockings or bandages) [48]. 

##### Compression Materials and Their Hemodynamic Effects

1. Inelastic materials

**Figure 3:**
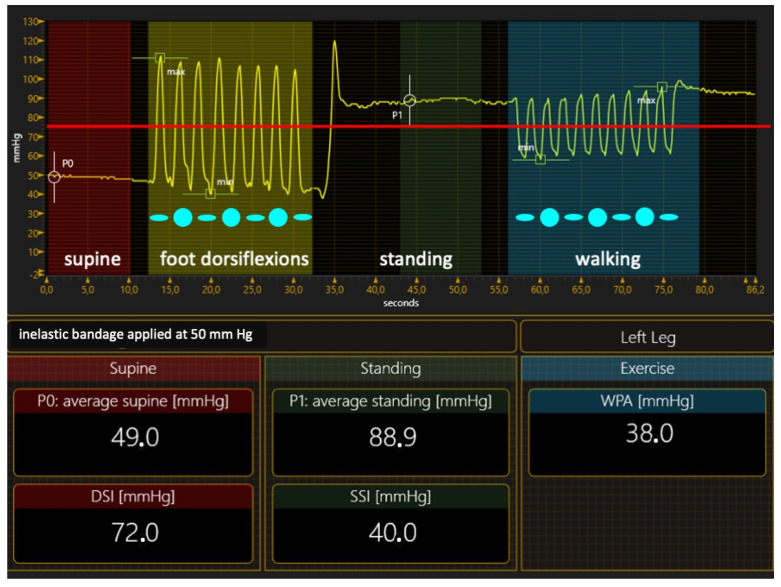
The pressure curve of an inelastic bandage exerts a compression pressure of 50 mm Hg. This pressure overcomes the intravenous pressure of about 75 mm Hg (red line) when performing foot dorsiflexion in a standing position and during walking, restoring a kind of valve mechanism; DSI—dynamic stiffness index; SSI—Static Stiffness Index; WPA—walking pressure amplitude.

Inelastic materials exert their effect by resisting the increase in the muscle volume during muscular contraction in a standing position and during physical activity (the leg will give way). When standing up from the lying-down position, inelastic material causes a significant compression pressure increase compared with supine pressure, and very high pressure peaks when walking (Figure 3). The main stiffness index, the Static Stiffness Index (SSI), which is the difference between the standing and supine pressure, is >10 mm Hg, which characterizes inelastic materials [49,50,51]. 

The strong standing pressure and the intermittently strong or very strong pressure peaks during exercise will overcome the intravenous pressure [52] intermittently occluding the vein, mimicking a kind of valve mechanism. Due to these effects, inelastic materials can significantly improve impaired venous hemodynamics by reducing venous reflux [53,54], increasing the calf pumping function [55,56], and decreasing the AVH [57].

Inelastic materials are challenging, and well-educated personnel are required when applying inelastic bandages. A series of papers demonstrated that only 10–60% of health personnel treating venous ulcers could apply the target pressure with different inelastic bandages [58,59,60,61,62,63]. This is different for adjustable compression wraps, which are easier to use and are self-applied, facilitating self-management by the patients [64,65,66].

2. Elastic materials

**Figure 4:**
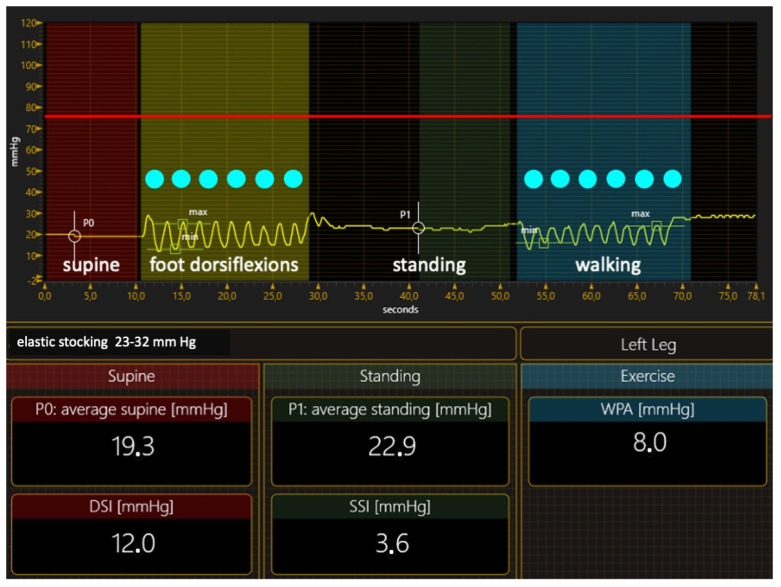
The pressure curve of an elastic stocking exerts a compression pressure of about 20 mm Hg. The pressure never approaches the intravenous pressure of about 75 mm Hg (red line) when performing foot dorsiflexion in a standing position during walking. Veins are not compressed. DSI—dynamic stiffness index; SSI—Static Stiffness Index; WPA—walking pressure amplitude.

The elastic materials (elastic stockings or elastic bandages with extensibilities higher than 100%) cannot achieve this intense pressure or pressure peaks during walking. Elastic material gives way to muscle expansion, which occurs during standing and physical activity, resulting in a very low pressure increase when moving from the supine to the standing position. The SSI is always < 10 mm Hg in the elastic range, and the walking pressure amplitude (WPA) and dynamic stiffness index (DSI) are very low too (Figure 4). 

**Figure 5:**
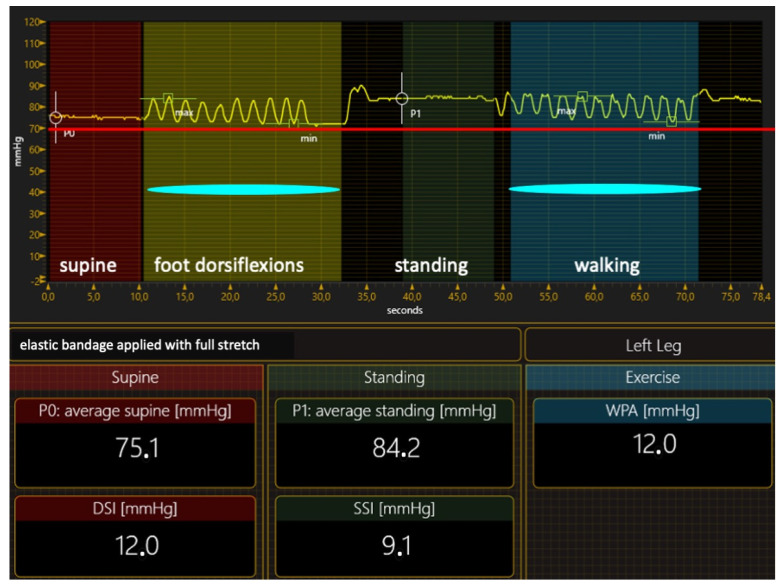
The pressure curve of an elastic bandage applied with full stretch exerts a compression pressure of about 75 mm Hg. Compression pressure is always the same or higher than the intravenous pressure (red line). Veins are always compressed. The bandage becomes painful within a short time. DSI—dynamic stiffness index; SSI—Static Stiffness Index; WPA—walking pressure amplitude.

Elastic materials tend to return to their initial length when stretched, and this “return force” is directly related to the stretch applied to the material. Suppose that the elastic material is strongly stretched to exert intense pressure (Figure 5), or several elastic stockings are superimposed. In this case, the final compression will be very painful and not tolerated by the patient after a short time from application. Consequently, when adequately stretched, an elastic bandage will exert a supine pressure of 30–40 mm Hg. Elastic stockings exert a wide range of pressure, from 18 to >50 mm Hg, according to their classification (I–IV class). Due to the very light pressure increase during standing and walking, the final pressure will never approach the intravenous pressure, resulting in the inability to narrow or occlude the veins [55], exerting a poor hemodynamic effect, which is significantly lower compared with inelastic material [56,57,58,59,60]. The advantage of elastic materials is that they are easy to apply and self-apply, usually as a single component. 

The hemodynamic effects of compression therapy are summarized in Table 4.

3. What material for ulcer treatment?

A long-standing debate exists about the preferred material used when facing a VLU. As described above, inelastic material (bandages or ACWs) is significantly more effective than elastic material in counteracting venous hemodynamic impairment [53,54,55,56,57]. As venous leg ulcers are due to a venous hemodynamic impairment, inelastic material, the best option to improve the venous hemodynamic impairment, should be the best treatment to increase the VLU healing rate. We have evidence that the higher the compression pressure, the higher the healing rate [11,67], which is in favor of inelastic bandages, which exert a much higher standing pressure than elastic materials.

- Inelastic bandages. When correctly applied to exert strong pressure, inelastic bandages can achieve an ulcer healing rate close to 100% in three months of treatment [68]. Therefore, preferring inelastic material in VLU treatment is the right option from a logical point of view, but we need ultimate proof that this is correct at present. In contrast, many papers claim the greater effectiveness of elastic stockings or bandages than inelastic material in achieving ulcer healing [69,70,71,72,73,74,75,76,77,78,79,80,81,82]. However, studies comparing elastic and inelastic devices have so many flaws that their conclusions are hard to believe [83]. Two significant deficiencies in papers on compression therapy must be highlighted: the lack of compression pressure measurement and stiffness assessment, and the lack of healthcare professionals’ expertise in applying inelastic bandages. The compression pressure, the dosage of the compression therapy, was seldom measured in the papers comparing different materials, even though compression pressure measurements are easy to perform [84]. Not measuring the compression pressure and not knowing the level of expertise of healthcare professionals [58] make it impossible to tell whether the bandages were correctly applied, making any conclusion challenging to trust. Inelastic bandages could also be so poorly applied that their compression pressure is lower than that exerted by an elastic kit [80]. In addition, not measuring the pressure or assessing the SSI produced a notable mistake in almost all studies comparing elastic and inelastic bandages. In these studies [69,70,71,72,73,74,75,76], the prototype of elastic material was the so-called four-layer bandage, which was dogmatically considered elastic, as it comprises four different elastic components. Nevertheless, measuring the supine and standing pressure of the final bandage and calculating its SSI showed that the SSI is in the inelastic range [85]. In conclusion, all these studies compared two different inelastic bandages, and the conclusions must be critically reviewed;
- Adjustable compression wraps (ACWs). These devices are quite inelastic and offer the advantages of inelastic material, already reported, in terms of improving the venous hemodynamics [86,87]. At the same time, they are straightforward to use and can be applied and re-adjusted by the patients themselves after a very short wear and education time [64,65,66]. ACWs have been proven more effective at achieving healing in patients with VLUs when compared with Unna boot bandages [88], four-layer bandages [89], or two-layer bandages [90]. Even though more studies must confirm these results, their outcomes seem promising;
- Elastic stockings. Is there any role for MCSs? As already reported [40], MCSs are very effective in preventing ulcer recurrence after ulcer healing, but they also have a role in the active VLU treatment, especially when using the so-called elastic kits of two superimposed stockings and exerting pressure at about 40 mm Hg. The elastic kits were effective in small ulcers of recent onset. They achieved ulcer healing in 36–96% of patients with these VLU characteristics [77,78,79,80,81]. As ACWs, elastic kits do not require expert personnel to be applied and allow self-management.

In conclusion, inelastic materials are the most effective treatment modality, and we must choose between inelastic composite bandages (difficult to apply) or Velcro^®^ devices, which are easier to apply and allow self-management, depending on personal expertise. Elastic kits may offer an alternative solution in small and recent-onset ulcers.

#### 4.1.2. Lifestyle and Physical Exercise

Patients suffering from venous ulcers are often elderly and not infrequently affected by comorbidities. The most frequent of these comorbidities are orthopedic impairment and obesity. Orthopedic impairment may prevent them from normal ambulation, leading to calf muscle pump inefficiency and reduced venous output from the lower leg. In addition, painful joints and obesity may favor long sitting positions. This condition results in sedentarism, the further deterioration of the venous stasis in the lower leg, and a vicious cycle sets in [91]. Encouraging an alternation of rest in a supine position with elevated legs, physical activity, weight loss, and compression garments is mandatory in these patients. Leg elevation aids venous drainage, reducing leg swelling and improving microcirculation. Leg elevation combined with wearing compression garments is so important that it increases the VLU healing speed and reduces the VLU recurrence rate [92,93]. Elevation was given an IA recommendation from the European Society of Vascular Surgery in 2015 [94].

Physical activity can be simple walking when the patients do not have a significant mobility impairment. Correct walking activates the calf muscle pump and increases the venous output from the lower leg. When the patients suffer from substantial joint impairment, an exercise program to improve mobility, and especially the range of motion of the ankle, knee, and hip joints, may favor VLU improvement and healing. At least these procedures may result in reduced venous ulcer recurrence rates [95]. In turn, it was shown that patients who kept on with orthostatism, became obese, or did not wear compression hosiery developed progressive venous symptoms that could expose them to an increased risk of VLUs [96].

Physical activity, including walking speed, duration, and ankle motion, is significantly reduced in patients with VLUs [97]. This is precisely contrary to what is necessary, as venous return from the lower leg is based on the venous pumping system requiring active calf muscle contraction and ankle mobility with a normal range of motion. Exercise programs focused on calf muscle activities and ankle rehabilitation are the most effective at increasing the VLU healing rate. 

Some reports demonstrated that patients adhering to exercise programs, specifically resistance exercise combined with ankle and/or foot range-of-motion exercises, as an adjunctive treatment to standard care, have a higher chance of healing and better functional outcomes than those who do not adhere to exercise programs [98,99,100,101]. In addition, they are also more likely to avoid an ulcer recurrence [95].

Although the evidence is still relatively weak (small studies, short intervention periods, etc.), at least we have some proof of the importance of physical exercise in improving the healing rate and preventing recurrences.

#### 4.1.3. Local Treatment

Most healthcare providers usually apply local dressings on the ulcer bed when treating patients with leg ulcers. They are claimed to create a moist environment on the ulcer that aids in faster healing. A moist environment makes removing debris or dead tissue from the ulcer bed easier. In addition, local dressings contribute to controlling exudate excess-retaining fluids from moving to peri-wound tissue to control inflammation and infections. 

Nevertheless, their effectiveness at increasing the VLU healing rate is still debated. A previous Cochrane review denied any role of local dressing in ulcer healing. The conclusion was as follows: “The type of dressing applied beneath compression has not been shown to affect ulcer healing” [102]. Only recently, a less undesirable conclusion more open to the positive effectiveness of local dressings was published by Cochrane reviewers: “In the light of the rest of the network meta-analysis evidence, we cannot be very confident about any conclusion, and the network as a whole represents low-certainty evidence” [103]. Even if data on VLU healing are not conclusive, it is possible to state that local dressings are, at least, effective in pain, infection, and exudate control, large ulcer healing, and cost reduction [104,105,106,107,108]. These effects may improve the patient’s quality of life [109].

#### 4.1.4. Pharmacologic Treatment

The effectiveness of systemic drugs at improving the VLU healing rate has been reported in several papers. Micronized purified flavonoid fraction (MPFF) is one of the most studied drugs in this field.

Flavonoids interact with several biological systems. They have an anti-inflammatory effect, inhibiting granulocyte and macrophage infiltration through the venous endothelium and reducing circulating growth factors and endothelial cell adhesion molecules. Finally, flavonoids prevent/reduce edema formation by decreasing capillary filtration [110,111,112]. The clinical beneficial effects of MPFF are due to these properties. Micronized purified flavonoid fraction as an adjunct to compression therapy increased the ulcer healing rate and shortened the healing time while improving venous symptoms in many randomized controlled trials (RCTs) [113,114,115,116].

Sulodexide, a drug combining low-molecular-weight heparin and dermatan sulfate with profibrinolytic and antithrombotic effects, was demonstrated to improve the VLU healing rate in a randomized multicentric placebo-controlled trial that enrolled 235 patients treated for three months [117]. More recent papers and meta-analyses confirm the effectiveness of sulodexide in VLU healing [118,119]. Also, a recent Cochrane review reports positive effects of sulodexide in VLU patients but also highlights that “the evidence is only low quality and the conclusion is likely to be affected by new research”, and that “Further rigorous, adequately powered RCTs examining the effects of sulodexide on healing, ulcer recurrence, quality of life and costs are necessary” [120].

Pentoxifylline inhibits neutrophil activation, cytokine-mediated white cell adhesion to the endothelium, and oxidative stress [121]. It was initially used in patients with peripheral arterial disease (PAD) [122] and in the off-label in VLUs. Pentoxifylline could improve VLU healing via some different mechanisms: it improves the microcirculatory blood flow by increasing the erythrocyte flexibility, induces prostacyclin synthesis, exerts a vasodilatory effect, inhibits platelet aggregation, and exerts a fibrinolytic effect [123]. The actual effectiveness of pentoxifylline at increasing the VLU healing rate is controversial. Some studies report good results for improving VLU healing compared with placebos [124], while others report conflicting results [125,126]. These contrasting data reflect different conclusions in review papers. While the Cochrane review states that “Pentoxifylline is an effective adjunct to compression bandaging for treating venous ulcers and may be effective in the absence of compression” [127], a more recent meta-analysis concludes that the “Currently available evidence suggests that pentoxifylline could help venous leg ulcers heal more quickly and effectively. However, the evidence is insufficient to prove the results due to moderate-certainty evidence. Large-scale, well-designed, randomized clinical trials are warranted” [128].

### 4.2. Invasive Treatment

#### 4.2.1. Surgical and Endovenous Treatment of Superficial Venous Insufficiency

The history of the surgical treatment of lower-limb varicosities goes back thousands of years. The first medical document describing ligation surgery and the surgical excision of varicosities was written by the Roman physician Celsus. It is followed by a description of the technique, like that of the contemporary saphenous stripping written by the Greek physician Galen, in which, for his technique, he described that varicosities were directly irritated by a hooked tool wire, aiming to extract as much of the vein as possible [129].

These methods are based on modern varicose vein treatment methods, such as ligation and stripping, as well as phlebectomy of the veins. Today’s varicose vein surgery is developing by leaps and bounds thanks to the basis obtained in ancient times.

The contemporary era of the surgical treatment of varicose vein disease began at the end of the nineteenth century when Friedrich Trendelenburg [130] described the great saphenous vein (GSV) ligation.

Today, there are two theories about the evolution of varicose veins and, accordingly, two main directions for their surgical treatment. The first one is descending theory, which proposes that varicose veins and chronic venous insufficiency are due to a failure of the proximal components of the superficial venous system: the valves at the saphenofemoral junction (SFJ) or saphenopopliteal junction (SPJ). According to this theory, treatment should begin with removing the SFJ or SPJ, abolishing venous reflux. Surgical methods such as high ligation, stripping, the ligation of varicose veins, and phlebectomy rely on this theory.

Until the first decade of the 21st century, the fundamental approach in the surgical treatment of varicose veins with great saphenous vein incompetence was long stripping [131] and its modifications.

This method has several variants: proximal stripping with a probe—the classical Babcock method; proximal stripping with the invagination (inversion) of the vessel; distal stripping with and without invagination [132,133,134]. These procedures have been performed safely for decades, although not always without associated morbidity. Postoperative pain, paresthesia due to saphenous nerve injury, hematoma, and bruising are the most common complications [135,136,137], which provoked the development of other methods, such as perforation imagination (PIN) stripping and cryo-stripping, in the hope of reducing postoperative complications [137,138,139,140]. Despite theoretically justified methods, the recurrence rate of varicose veins after five years has been reported to vary from 20% to 80% [141].

In the past decade, alternative treatments, such as endoluminal procedures, have gained popularity. These procedures can be divided into two groups: thermal procedures, in which the ablation of the veins is achieved via heat, and non-thermal procedures, in which the veins are obliterated chemically after the mechanical injuring of the vein wall by the tip of the catheter. 

The most widely used endoluminal procedures are radiofrequency ablation (RFA) and endovenous laser ablation (EVLA). The RFA and EVLA procedures were introduced at the end of the 20th century. In contrast to stripping operations, the great advantage of endoluminal methods is that they are minimally invasive. Treated patients can undertake normal physical activities the same or next day, often including sports, and, as a rule, with no impairment due to the previous endovenous operation [142]. In radiofrequency procedures, the veins are heated to 120 °C in sections by a radiofrequency probe inserted through the lumen of the catheter [143]; for laser procedures, the use of high water absorption wavelengths (1470–1940 nm) and radially emitting laser probes is preferred. The temperature reached by a laser with a wavelength of 1470 nm with a radial probe is about 120–140 °C (±20 °C) [144,145]; it is now possible to precisely occlude a saphenofemoral recurrence with a remaining saphenofemoral stump [146]

Other new endoluminal methods are the non-thermal procedures: mechanochemical ablation (MOCA) and the cyanoacrylate (adhesive) closure system.

The MOCA procedure is used to seal a vein via a combination of mechanical action (the tip of the catheter rotates or has flexible cutting elements, resulting in painless damage to the intimate layer of the vein wall) and chemical action (a sclerosing agent is injected into the vein below the tip of the catheter) [147,148]. In the adhesive system, the vein is sealed with a special vein-adhesive material (mono-cyanoacrylate) [149].

Usually, all open surgical and endoluminal methods are completed with the mini phlebectomy of varicosities introduced by Muller [150] and supplemented by Varady hooks [151].

Based on descending theory, all these methods either remove or destroy the great saphenous (GSV) or short saphenous (SSV) vein to eliminate reflux in the superficial venous system. In addition to these methods, alternative techniques exist, relying on the ascending theory of varicosity, which advocates the proximal progression of distal venous incompetence and is supported by the observation that superficial venous incompetence often occurs with a competent saphenous junction. CHIVA (Ambulatory Conservative Hemodynamic Treatment of Venous Insufficiency) and ASVAL (Ambulatory Selective Varices Ablation Under Local Anesthesia) are two techniques characterized by the targeted therapy of the venous system without saphenous trunk destruction or removal [152].

CHIVA is the hemostatic column of blood fragmentation, achieved via the selective ligation of the truncal vein, the preservation of communicating branches draining into the deep venous system, and the ligation of refluxing saphenofemoral connections [152]. 

According to a review published by Cochrane in 2013, CHIVA reduced recurrence rates, improved quality-of-life results, and reduced side effects compared to stripping and compression [153]. A comparison between CHIVA, stripping, and EVLA found that CHIVA and EVLA patients had improved cosmetic results and reduced pain scores compared to those undergoing open surgery [154].

ASVAL is a minimally invasive technique based on the ascending theory of varicosity development. ASVAL aims to remove varicose tributaries, considered to be the origin of incompetence, without treating the saphenous trunk. The removal of varicosities reduces the hemodynamic load on the saphenous vein [155].

A retrospective analysis comparing the results of ASVAL and saphenous vein sparing revealed a reduction in more than two-thirds of cases over a four-year follow-up period, while symptoms improved or disappeared in most cases. There was no recurrence in 95% of patients at one year nor in 88.5% of patients at four years [156].

**Figure 6:**
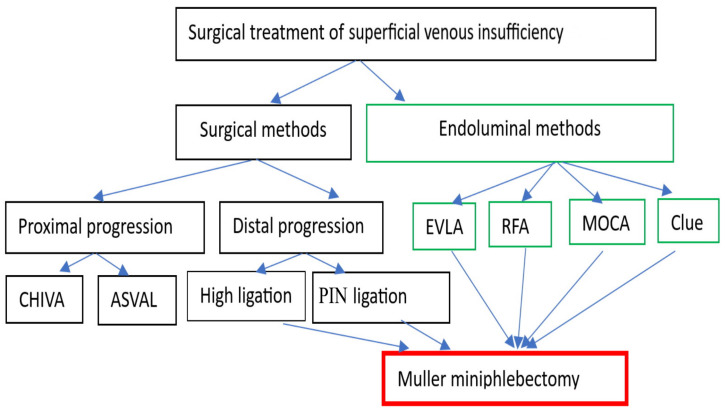
Invasive methods of superficial venous insufficiency treatment (CHIVA—Ambulatory Conservative Hemodynamic Treatment of Venous Insufficiency; ASVAL—Ambulatory Selective Varices Ablation under Local Anesthesia; PIN—perforation imagination; EVLA—endovenous laser ablation; RFA—radiofrequency ablation; MOCA—mechanochemical ablation).

The summary of the invasive treatment of superficial venous insufficiency is presented in Figure 6.

In conclusion, the surgery for varicose veins is still evolving. There has yet to be a consensus on which treatment modality is the gold standard, and large multicenter studies are needed to prove the benefit of one of these surgical modalities in treating chronic venous insufficiency.

#### 4.2.2. Surgical and Endovenous Treatment of Deep-Vein Occlusion

The main venous outflow of the lower limb is the deep venous system [157], and the foremost cause of vein occlusion syndromes is deep venous thrombosis (DVT), where the DVT of the iliofemoral segment is more clinically significant, such as iliofemoral DVT, which is associated with a higher risk for recurrent venous thromboembolism (VTE) and a higher risk for the development of a post-thrombotic syndrome (PTS) [158]. Other causes of deep-vein occlusion syndromes are anatomical conditions, such as the compression of the left common iliac vein between the right common iliac artery and the fifth lumbar vertebra, also known as May–Thurner syndrome [159], which causes non-thrombotic deep-vein occlusion syndromes. Unfortunately, May–Thurner syndrome causes non-thrombotic deep-vein occlusion syndromes and is also the source of iliofemoral DVTs [160]. 

Usually, the surgery of deep venous systems is the most challenging part of the work of a vascular surgeon, such that the results of surgical procedures in the deep venous system are worse than those in the arterial system. 

**Figure 7:**
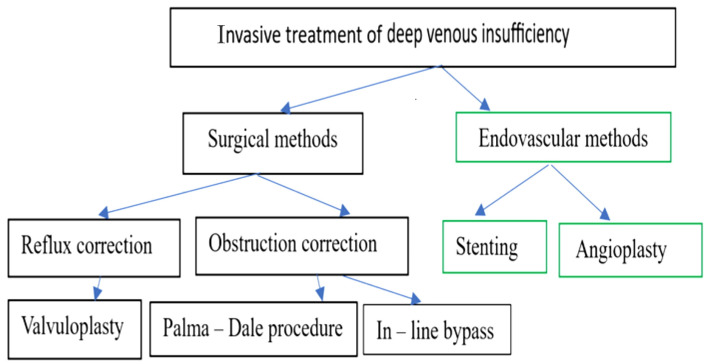
Invasive treatment of deep venous insufficiency.

Surgical procedures in the deep venous system are divided into two directions depending on the cause of the venous insufficiency: interventions to correct venous reflux and interventions to correct venous obstructions; both are consequences of PTS (Figure 7). 

In turn, venous reflux surgery depends on the condition of the valve. For example, performing a valvuloplasty and a valve transposition in cases of intact but incompetent valves is better. Still, in the case of destroyed valves, an axillary vein autotransplantation is the best choice [161].

Dr. Kistner performed the first open valvuloplasty in 1968 [162], and, today, there are a few types of valvuloplasty: internal valvuloplasty, external valvuloplasty, and transcommissural valvuloplasty [163].

The most famous surgical procedure for deep venous obstruction is the crossover bypass (Palma–Dale procedure) [164,165]. This procedure is performed when it is impossible to recanalize the occluded area or with extended lesions of the iliofemoral segment. In a crossover bypass, the great saphenous vein of a donor limb is exposed and implanted into the opposite saphenofemoral junction. If it is not possible to use the GSV, then a 10 mm PTFE (polytetrafluoroethylene) femoro-femoral bypass graft can be used. Another option is an in-line bypass procedure, which is indicated in the long femoro-iliocaval occlusive segment using an expanded PTFE graft, the most used material for bypass. An additional arteriovenous fistula is created distally to maintain inflow, and it is closed 4–8 weeks after surgery [166]. Unfortunately, life-long anticoagulation is usually needed. When it is not possible to perform crossover or in-line procedures, an endo-phlebectomy is the last hope, in which the post-thrombotic vein is longitudinally exposed at various segments, and the synechiae attached to the intimal layer is carefully removed with scissors. 

The treatment for deep-vein occlusion syndromes changed in the 1990s with the introduction of endovascular techniques. In the past, open surgery, including a bypass for chronic venous occlusive disease and thrombectomy for acute thrombosis, were invasive procedures, often used unselectively without proper pre- and intra-operative imaging and frequently with disappointing results. These techniques have been widely replaced by endovascular interventions, such as percutaneous recanalization combined with stenting [167].

**Figure 8:**
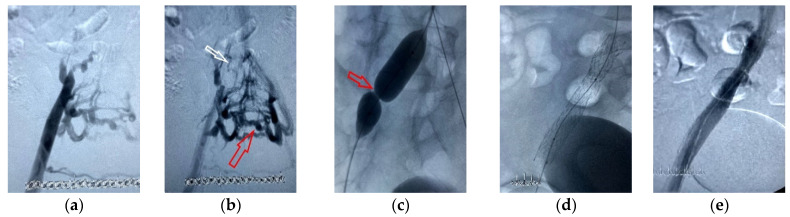
Endovascular treatment of the occlusion of the iliac veins. (**a**,**b**) Right iliac phlebography shows occlusion of right common iliac vein (CIV) (white arrow) and severe collaterals from right internal iliac vein to contralateral iliac veins (red arrow); (**c**) balloon dilatation after recanalization of occluded part of right CIV (red arrow shows strong resistance due to scarring in the vein, 15 atm pressure was given); (**d**) right CIV and external iliac vein (EIV) is stented; (**e**) phlebography after stenting—vein inflow from CIV restored, all collateral veins disappeared.

Over the past decade, insights into treating deep venous disorders via stents have increased. Venous stenting is recognized as a possible treatment to help assist patients with symptomatic vein occlusion syndromes as an alternative to conventional surgery (Figure 8). 

Treating deep-vein occlusion syndromes via stents, including involvement in the inferior vena cava, has acceptable patency rates and reduced symptoms in most patients [168]. In a published study with over 1500 patients, iliac vein stenting for vein occlusion syndromes was considered safe, with high patency rates of up to five years. Improvements in pain, ulceration, and limb swelling were noted [169]. A follow-up meta-analysis of 37 studies demonstrates good technical success, with minimal periprocedural complications and symptom relief at the final follow-up for five years. Complication rates are less than 1% for major bleeding, pulmonary embolism, and mortality, with 1.0–6.8% for early stent thrombosis [170]. 

The endovascular treatment of the occlusion of the iliac vein is presented in Figure 8.

Most initial venous stents were not designed for use in the venous system. Initial stent development addressed these challenges by increasing the crush resistance needed in venous stents [171]. Recently, dedicated venous stents were introduced, which, in theory, with increased chronic outward force and radial resistive force, should have better results than non-dedicated stents; today, we have a few works confirming that dedicated stents have results comparable to those for non-dedicated stents in terms of clinical outcomes, QOL improvement, and stent patency [172]. What about vein balloon angioplasty alone? The research work published by Aurshina A et al. in 2019 shows that in 49% of procedures, balloon dilatation relieved a previously stenotic area, and there was a highly variable response to balloon venoplasty, with 22% of procedures demonstrating no change and 29% of procedures resulting in a worsening of the cross-sectional area. This decrease in the area could be explained by the elastic recoil or spasm of the vessel wall upon dilatation. Regardless of the etiology, these require routine balloon dilatation before stent placement [173].

In conclusion, endovascular treatment, especially the stenting of the iliofemoral segment, has become an essential treatment option for chronic (thrombotic or non-thrombotic) obstructive venous disease. All vein occlusion syndromes should be treated with stenting because of the unintended chance of recoil and spasm. Balloon dilatation alone will not eliminate the external compression or PTS occlusions responsible for these lesions.

#### 4.2.3. Surgical Debridement and Skin Grafting

The direct surgical treatment of venous ulcers involves surgical debridement and skin grafting. Regular debridement needs to reduce the necrotic burden and achieve healthy granulation tissue [174]. The debridement can be performed with sharp surgical, enzymatic, mechanical, autolytic, and chemical techniques. Surgical debridement is usually performed in the operating room with systematic or regional anesthesia by removing slough and necrotic tissue [175]. Wounds close by two main mechanisms: epithelial migration and wound contraction. Both processes occur from the edges of the wound and aim to fill the lesion and shrink the wound edges. Unfortunately, these natural mechanisms are not always sufficient. Skin grafts or flaps can be considered alternative solutions to accelerate wound healing [176]. Early skin grafting significantly improves quality of life by reducing pain and accelerating wound healing. 

There are two types of skin grafts: split-thickness skin grafts (STSGs) and full-thickness skin grafts (FTSGs). Split-thickness skin grafts consist of the epidermis and some layers of the dermis. Full-thickness skin grafts are composed of the epidermis, dermis, and various layers of subcutaneous tissue [176]. STSGs are characterized by a poor cosmetic outcome, but they require lesser revascularization after implantation, which is why these grafts can be used to treat wounds with damaged blood supplies, such as venous insufficiency ulcers [177]. If the most crucial goal is the aesthetic outcome, then FTSGs are the best choice. 

### 4.3. Challenges in the Treatment of Mixed Arterial–Venous Leg Ulcers

The coexistence of both venous and arterial diseases has been reported in 15–26% of patients with lower-extremity ulceration. Also, arterial insufficiency (ABI < 0.8) is postulated to be present in up to 30% of the patients with VLUs [178,179,180,181]. 

Discovering arterial disease in patients with VLUs is crucial because compression therapy is the primary treatment for VLUs and, simultaneously, it would be challenging in patients with PAD due to the probability of worsening in critical limb ischemia cases [182]. Patients with mixed arterial–venous ulcers may complain of more leg pain than those with venous ulcers alone [183]. Furthermore, the inadequate presence of granulation tissue and rolled wound edges of VLUs could be the contemporary signs of arterial insufficiency [183,184]. However, ABI would be determinative of PAD diagnosis, especially in old patients who may not have claudication because of decreased mobility. In addition, because the existence of pulsation cannot rule out the possibility of PAD, ABI should be performed for all patients who complain of VLUs [182,183,184]. 

Until recently, the optimal treatment for mixed arterial–venous ulcers remained unclear. However, compression therapy with a pressure of 20–30 mmHg would be acceptable in cases with ABIs between 0.5 and 0.8 to avoid the deterioration of the tissue vitality [185,186]. Patients with ABIs less than 0.5 should not receive compression therapy before any revascularization procedures [187,188]. 

## 5. Focus on Prevention: What More Can We Do?

### 5.1. Primary Prevention and Risk Factors

The risk factors for developing a first-time VLU need to be better investigated, and primary prevention in patients with CVD needs to be more appreciated in general practice. A systematic review identified several risk factors in developing a first VLU: older age, deep-vein reflux, DVT, higher body mass index (BMI), low physical activity, arterial hypertension, and family VLUs [189].

During the clinical examination, the ulcer surroundings and skin changes, such as corona phlebectatica paraplantaris, varicose veins, or signs of chronic skin inflammation, should be documented [190,191,192]. Hemosiderosis, lipodermatosclerosis, stasis dermatitis, atrophie blanche, and corona phlebectatica paraplantaris are considered to have predictive value for the development of VLUs [193] and/or the recurrence rate [194]. Of particular clinical relevance is the corona phlebectatica paraplantaris, as this can be an early isolated clinical sign of severe CVD [195]. The venous plexus of the foot does not carry venous valves; therefore, venous ectasia manifests early here in venous hypertension. The CEAP classification of CVD, updated in 2020 and established internationally, includes this aspect by adapting the clinical severity score (C4c: corona phlebectatica paraplantaris) [196].

A higher BMI and low physical activity indicate that lifestyle factors are also involved in VLU development. The risk factor “obesity in VLU” comprises several aspects. In addition to the limited general and ankle mobility of some obese patients, obesity directly influences venous hemodynamics by raising the intra-abdominal pressure. In these situations, the underlying CVD has a greater impact, increasing the risk of VLU recurrence and decreasing the ulcer healing rate [192,197,198,199]. Scholl et al. published a case series of patients who developed VLUs exclusively due to obesity-associated CVD without any venous pathology [200]. Several studies have shown that obesity correlates with increased intra-abdominal pressure and a concomitant increase in pressure in the external iliac and femoral veins, resulting in a reduced flow velocity in the femoral vein [201,202,203,204]. The altered pressure and flow characteristics lead to venous hypertension in the lower extremities. Reduced venous flow velocity, in turn, triggers the CVD-associated inflammatory response. 

Moreover, obesity significantly impacts the development of VLUs through adipose tissue mass dysfunction by disrupting the microcirculation caused by the increased secretion of pro-inflammatory cytokines [198,205]. This pathophysiological relationship probably explains the association of the severity of post-thrombotic syndrome with the degree of obesity [206].

Although, as mentioned above, physical exercise, by increasing the movement of the ankle joint and strengthening the muscle pump in the calf of the leg, may help prevent the worsening of the disease [95,96]. The Cochrane meta-analysis published in 2016 and updated in 2023 showed that there is currently insufficient evidence to assess the benefits and harms of physical exercise in non-ulcerated CVI subjects. Therefore, the authors concluded that further research into the effect of physical activity considering the types of exercise protocols (intensity, frequency, and time), sample size, blinding, and homogeneity according to the severity of the disease is required [207].

Therefore, regarding primary prevention, it is essential to consider the risk factors and include them in the treatment strategies.

### 5.2. Recurrence Prevention

People with VLUs often live with a lifelong ulceration, healing, and recurrence cycle. As over 50% of venous leg ulcers will recur within 12 months of healing, a comprehensive knowledge of the risk factors associated with recurrence is required by health professionals caring for persons with VLUs [208,209,210].

Several randomized trials have shown that invasive therapy of the underlying superficial-vein insufficiency accelerates VLU healing [43,211,212] and reduces the incidence of recurrence [42,213]. These interventions can be endovenous (“ablative”) or open surgery (“surgical”). Even in patients with simultaneous segmental reflux in the deep venous system, the recurrence rates for VLUs were significantly lower in the intervention group (24%) compared to the compression group (52%) [42]. With the increasing severity of deep-vein insufficiency, the influence of invasive interventions on the recurrence rate becomes less, and the effect of the intervention is no longer statistically significant. Nevertheless, pre-existing reflux in the deep venous system should not be considered a contraindication for the invasive therapy of underlying superficial venous reflux because the intervention may improve CVD-associated symptoms [214,215].

Case series on peri-ulcerous ultrasound-guided foam sclerotherapy [216,217] showed a low recurrence rate in addition to the accelerated healing of the ulcerations [218]. Randomized controlled study results still need to be carried out.

So far, data with long-term follow-up after recanalizing the deep venous system in post-thrombotic syndrome still need to be included. Venous stenting as a strategy for preventing VLUs may be useful in selected individual cases [45].

In addition to the early invasive treatment of the underlying pathology in the superficial and/or deep venous system, continued medical compression therapy prevents VLU recurrence. A prospective controlled randomized trial investigated the effect of compression hosiery (ankle pressure of 35–45 mmHg) in 153 patients. The rate of VLU recurrence was significantly reduced from 54% in the compression group compared to 24% in the placebo group [219]. However, three other randomized controlled trials comparing different compression classes of hosiery showed no significant differences [40,41,214]. When selecting the compression class, it is crucial to consider that a higher compression class may be associated with a decrease in adherence [41,215,219,220]. In turn, poor adherence to medical compression therapy causes a higher rate of VLU recurrence [40,214,221]. Studies on the wearing time in the prevention of recurrence are required. In clinical practice, the recommendation is to wear the compression hosiery as consistently as possible whenever the legs are not elevated.

Some of the comorbidities associated with CVD (mentioned above) are also risk factors for a more severe course of CVD and negatively affect the healing process and increase the risk of recurrence [189].

Other relevant risk factors for recurrence are a long history of VLUs, a large wound area, obesity, limited mobility in the upper ankle, lack of patient education, lack of self-management, and lack of treatment adherence. These risk factors should be minimized preventively [42,100,204,213,222,223,224]. The positive effect of education on healing in VLUs has been shown in several studies [100,224,225,226]. The main reason is that structured education increases adherence. Adherence is essential for therapeutic success, especially in compression therapy [224]. Studies investigating a correlation between education and recurrence prevention have yet to be made available. However, it can be postulated from the available data that education increases adherence, thereby improving healing and preventing recurrences. Patients with healed VLUs may benefit from educational actions that include information on risk factors and long-term prevention strategies.

Physical activity and exercises should be recommended to patients with healed VLUs to improve venous return, strengthen the calf muscle pump, and increase the range of motion in the ankle joint [227,228]. A specification of suitable exercises is not possible due to the limited data. Activities should be customized to the patient’s physical capabilities. 

Data on whether weight reduction in obesity reduces the recurrence rate of VLU are few. However, because severe comorbidities coexist in these patients, the correlation with the worsening of pre-existing CVD due to the obesity-related increase in the intra-abdominal pressure and the secretion of proinflammatory cytokines, it seems reasonable to recommend weight normalization in patients with VLUs and concomitant obesity. 

VLU recurrence prevention involves a combination of strategies, including early interventions addressing underlying venous insufficiency, compression therapy, lifestyle modification, education, and the monitoring of early signs of recurrence by patients and health professionals. 

## 6. Conclusions

A VLU-free world is an ambitious aim, but it may be challenging to achieve with the current knowledge of the pathophysiology and diagnostic, and therapeutical protocols. Conservative and invasive procedures, when properly prescribed, will undoubtedly help. Compression therapy and lifestyle adaptations may play significant roles in conventional approaches, but local and general treatment must be considered. 

To decrease the incidence of VLUs, the long-term goal must be to identify high-risk patients early and initiate appropriate preventive measures. Patients with varicose veins are at a significantly increased risk of ulceration, especially those with skin changes of chronic venous insufficiency and associated deep-vein incompetence. Additionally, CVI is often not diagnosed until VLUs develop. Its treatment can be complex and challenging at this stage, often leading to patients experiencing repeated ulceration, healing, and recurrence patterns. 

In the meantime, a joint effort of all the active stakeholders to provide a standard and universally acceptable indication with coordinated initiatives to prevent VLUs everywhere is essential, incorporating suggestions that consider the feasibility linked to countries’ different geographies and socioeconomics worldwide.

## Figures and Tables

**Table 1:** Diagnostics of leg ulcers.

**Medical History**	
**History of present symptoms and signs**	**Duration and presence of symptoms:** Cramps, tired legs, swollen legs, heavy legs, restless legs, venous claudication, itching Pain: distribution, intensity (VAS score 0–10), duration, intermittent, during night/day, pain during dressing changes
	**Duration and presence of signs:** Varicose veins: duration, uni-/bilateral, bleeding from the vein Swelling: uni-/bilateral, region: around the ankle, whole leg, relation to standing/sitting a whole day Active ulcer: spontaneous/post-traumatic, duration, dressings (type/frequency of changes), compression therapy
**Past signs**	Previously healed/recurrent ulcers: spontaneous/post-traumatic, duration, dressings, compression therapy DVT/SVT/PE: time of occurrence, therapy Previous leg fractures Previous surgical therapy
**Comorbidities**	Diabetes mellitus, hypertension, chronic renal insufficiency, heart failures, malignancy, rheumatoid arthritis, PAD, obesity, back problems
**Treatment**	Treatment of present and past varicose veins: laser, sclerotherapy, surgical treatment, endovenous ablation (non-thermal/thermal), compression therapy (short-/long-stretch bandages, stockings, Velcro^®^ materials) Treatment of present ulcer: dressings, therapy of surrounding skin, compression; where and by whom treatment is provided (patient/nurse/in hospital/in healthcare center); medications (anticoagulants, contraceptives, hormone replacement therapy, antidiabetics antihypertensives, immunosuppressive therapy, other)
**Allergies**	Contact/systemic drug reaction
**Pregnancy**	When, number, signs, and symptoms during pregnancy, therapy of signs/symptoms
**Family history**	Presence of varicose veins in relatives, ulcers, DVT
**Occupation**	Prolonged standing/sitting
**Bad habits**	Smoking, alcohol consumption, drug use
**Trauma**	Mechanical, chemical, radiotherapy, chemotherapy, etc.
**Clinical Assessment**	
**Inspection and palpation**	Mobility, BMI Presence of varicose veins, corona phlebectatica Limb swelling: Stemmer’s sign, non-pitting/pitting, Bisgaard sign Skin changes: hyperpigmentations/redness (whole leg/during the vein, eczema), lipodermatosclerosis/atrophie blanche Peripheral arterial pulses, capillarity refilling Groin lymph nodes Leg temperature (cold/warm) Scars after previous surgical therapy, trauma Trophic changes in nails
	Leg ulcers: where, number, size, wound bed (necrosis, fibrin (ogen), granulation tissue, epithelial tissue, isles in wound bed), edges, surrounding skin, smell, presence of infection, wound exudate, possibility of ankle movements
**Functional/Diagnostics Testing**	
**Venous system**	CW Doppler: S–F junction reflux Duplex US Photoplethysmography
**Arterial system**	Measurement of ABI (with CW Doppler; automatic)
**Lymphatic system**	Limb circumferences, perimetry, bioimpedance
**Non-invasive/invasive tests**	Monofilament test Capillaroscopy Venography IVUS Angiography Lymphoscintigraphy CT MR
**Microbiological**	Swab for pathogenic bacteria and fungi
**Skin/ulcer biopsy**	Pathohistological examination Direct immunofluorescence
**Blood tests**	Complete/differential blood count, C-reactive protein, erythrocyte sedimentation rate, blood glucose, HBA1c, blood lipids electrolytes, urea, creatinine, liver function tests tests of coagulations total proteins, circulating immune complex, immunoglobulins, cryoglobulins, APC resistance, protein C, S, homocysteine ANAs, ENA, anti-DNA, ANCAs, antiphospholipid antibodies, lupus antibodies, pemphigus and pemphigoid antibodies, vitamins (B12, D3, folic acid, A), trace elements (Fe, Zn, Mg, Cu) Serological tests (lues tests—TPHA, leprosis, tbc)

VAS—visual analog scale for pain; DVT—deep-vein thrombosis; SVT—superficial-vein thrombosis; PE—pulmonary embolism; PAD—peripheral arterial disease; BMI—body mass index; CW Doppler—continuous-wave Doppler; S–F junction—saphenofemoral junction; Duplex US—duplex ultrasound; IVUS—intravascular ultrasound; CT—computed tomography; MR—magnetic resonance; HBA1c—hemoglobin A1c, glycated hemoglobin; APC—activated protein C; ANA—antinuclear antibodies; ENA—extractable nuclear antigen; anti-DNA—anti-double-stranded DNA (anti-dsDNA); ANCAs—antineutrophil cytoplasmic antibodies; Fe—ferrum; Zn—zinc; Mg—magnesium; Cu—copper; TPHA—Treponema Pallidum Hemagglutination Assay; tbc—tuberculosis.

**Table 2:** Assessment of leg ulcer.

The Questions We Ask Ourselves	The Causes/Symptoms/Signs	Tests for Making the Diagnosis
**What is the immediate cause of the wound?**	- Trauma - Too-tight compression - Spontaneous	
**Is there any underlying pathology?**	- Venous disease: vein symptoms: varicose veins; mild ankle swelling during the day; hyperpigmentation; corona phlebectatica; lipodermatosclerosis; stasis eczema; atrophie blanche; past DVT/SVT; varicose veins/ulcer in family; reflux at SF junctions; insufficient veins at DU investigations; venography - Arterial disease: intermittent claudication; rest pain; poor perfusion (loss of pedal pulses, cold foot, pale skin); atrophic skin; loss of hair on the leg; trophic changes or gangrene; necrosis on the wound bed - Mixed venous–arterial etiology - Diabetic leg ulcer: neuropathic: warm, sensory deficit, diminished sweating, dry skin, motor neuropathy with a high arch and claw toes, Charcot foot; ischemic: pale, cold, absence of pulses, intermittent claudication; neuro-ischemic foot - Vasculitis: pain ulcers; small ulcers with necrosis - Malignancy: usually no pain; hypertrophic ulcers - Infection: high body temperature, redness around the ulcer - Bullous dermatosis - Lymphoedema stage III: primary or secondary cause lymphoedema: papillomatosis, Stemmer’s sign, non-pitting/pitting edema	- Reflux at SF junctions, insufficient veins at DU investigations, plethysmography, venography - Blood lipids, low ABI (below 0.85 or below 0.5), arteriography - Blood glucose, HBA1c, monofilament test, capillaroscopy, ABI - ANCA, biopsy (HPE, DIF) - Biopsy - Swab, CRP, ESR, complete/differential blood count - Pemphigus and pemphigoid antibodies, biopsy - Limb circumferences, perimetry, bioimpedance, lymphoscintigraphy
**Does the patient have any (medical) conditions?**	Diabetes mellitus, malnutrition, cardiovascular disease, anemia, renal disease, rheumatoid arthritis, cerebrovascular disease, old age, reduction in sensory perception, increasing susceptibility to trauma, therapy with immunosuppression agents, malignancies and their treatment, smoking, alcohol consumption, drug use, prolonged standing/sitting, obesity, poor mobility	

SF−saphenofemoral; DU—Doppler ultrasound; DVT/SVT−deep-vein thrombosis/superficial-vein thrombosis; ABI−ankle–brachial index; HBA1c—hemoglobin A1c, glycated hemoglobin; ANCAs−antineutrophil cytoplasmic antibodies; HPE−histopathological examination; DIF−direct immunofluorescence microscopy; CRP−C-reactive protein; ESR−erythrocyte sedimentation rate.

**Table 3:** Recommendations of investigations in patients with venous leg ulcers/level of evidence.

Palpation of lower-extremity arterial pulses and calculated ABI are recommended for all patients with suspected venous leg ulcers	C
Duplex ultrasound sonography is recommended for patients with venous leg ulcers to assess venous reflux and/or obstruction	C
Biopsy is recommended for patients with venous leg ulcers if healing stalls	C
Biopsy is recommended for patients with ulcers if there is suspicion that the ulcer may be venous, but it has an atypical appearance	C
Referral to a subspecialist is recommended for patients with venous leg ulcers if healing stalls	C
Referral to a subspecialist is recommended for patients with ulcers if there is suspicion that the ulcer is not venous, but it is of an atypical appearance	C
Screening of patients using a hand-held Doppler detector makes sense only in mild involvement when only telangiectasias and venectasias are present	C
X-ray contrast venography, magnetic resonance, or computed tomography venography are reasonable to perform only in a small number of selected patients who have anatomical venous anomalies, and in those patients in whom surgical intervention on the deep venous system is planned.	C

ABI−ankle–brachial index; C−based on expert opinion and consensus guidelines in the absence of clinical trials.

**Table 4:** Hemodynamic effects of compression therapy.

	Venous Reflux (VR)	Calf Pumping Function (CPF)	Ambulatory Venous Hypertension (AVH)
**Elastic material**	~	~	~
**Inelastic material**	+++	+++	+++

~ Very low influence; +++ high influence.

## Data Availability

We used PubMed and Web of Science to screen articles for this narrative review. We did not report any data.
